# A review of HIV prevention among young injecting drug users: A guide for researchers

**DOI:** 10.1186/1477-7517-2-5

**Published:** 2005-03-17

**Authors:** Kate A Dolan, Heather Niven

**Affiliations:** 1 The Program of International Research and Training National Drug and Alcohol Research Centre The University of New South Wales, Sydney, Australia

## Abstract

Young people aged 15–24 years account for fifty percent of all new AIDS cases worldwide. Moreover, half of all new HIV infections are associated with injection drug use. The average age for initiation into injecting drug use is 20 years of age. This paper investigates whether HIV prevention programs have reduced risk behaviours in young people.

## Introduction

Young people are at the forefront of the HIV/AIDS epidemic as it continues to spread worldwide. An estimated 12 million people aged between 15 and 24 years are living with HIV or AIDS around the world. Of the five million new HIV infections in 2001, over half were among youth aged 15–24 [1]. Six thousand young people become infected with HIV every day, and over half of all new HIV infections are related to injecting drug use [1]. In some regions, such as Eastern Europe and Central Asia, nearly all reported HIV infections are linked to drug injection, the majority being young injectors. In some developing and transitional countries, injection drug use is spreading rapidly and the age of initiation of drug injecting is decreasing [2].

Adolescence is an age when critical health behaviours are established, including behaviours related to sex and drug use (Ball, 2000). Most of these behaviors can be predicted from the risk environment, with clusters of risk behaviour being common, such as alcohol abuse and unprotected sex, particularly among marginalised and vulnerable youth. There is some evidence that young injectors think and behave differently to older IDUs and are treated differently within their communities. Specifically, young IDUs have less knowledge about HIV/AIDS, have a lower perception of their risk of acquiring HIV through either drug injecting or sex, and are less likely to identify as being an IDU than older IDUs [3].

Moreover youth have a heightened risk of HIV infection as a result of many factors, including risky sexual behaviour, substance abuse (including injecting drug use), and lack of access to HIV information and prevention services. It is crucial that barriers to accessing services that youth face are recognized by youth health services, including programs to prevent HIV infection. Marginalized young people, including homeless youth and ethnic minorities, may be at an heightened risk due to factors such as stigma (which may prevent access to critical HIV/AIDS information and prevention programs), pressure to engage in unprotected sex in exchange for food, shelter or money and the use of illicit drugs. In an attempt to minimize the HIV epidemic, a range of HIV interventions have been developed [1]. These interventions are designed to change behaviours of individuals who are at risk of acquiring or transmitting HIV infection.

Programs for young people offer the greatest potential for changing the course of the epidemic [1]. However, research into HIV prevention in youth is an area that has seemingly been neglected, as most studies focus on adult populations. This remains the case despite global findings that injecting commences during adolescence [4]. Researchers need to redress this neglect of youth if they are to produce evidence necessary to allow an effective global health response.

Comparison of programs for effectiveness will allow relative judgments to be made regarding effectiveness, and factors such as cost effectiveness can be taken into account to assist in the allocation of resources, particularly in resource-poor settings.

Calls for international standardisation have appeared in the literature since at least 1999 [5]. According to Suishansian et al [6] the need for standardisation, collection, interpretation, and integration of program monitoring data with biological and behavioural surveillance data on HIV/AIDS associated with IDU is critical to informing and guiding appropriate prevention responses.

The aim of this paper is to review recent literature of HIV prevention programs for young injecting drug users (IDUs) in an attempt to establish whether this call for standardisation has been heeded by researchers and program managers alike. Recommendations for improvement in evaluations to allow comparisons is provided, to assist in informing policy and program managers in the development of evaluation designs.

## Method

This review of the literature involved assessing the effectiveness of HIV prevention programs for young, and new injecting drugs users. The review also included programs undertaken to prevent initiation of drug injecting and transition from non-injecting to injecting drug use.

Databases such as Medline, Psychinfo, Web of Science, Sociofiles, ERIC, Psychofiles and Aidsline were searched. International and local websites of drug addiction and prevention services or agencies and AIDS agencies and libraries were examined. A similar search strategy was also used to cover the grey literature. Unpublished literature, such as conference presentations and agency reports, was drawn from a number of different searches conducted on the worldwide web.

Nonetheless, in order to evaluate the efficacy of the HIV programs, only those that provided information regarding the effectiveness of the outcome were included. Not surprisingly, this somewhat restricted the number of studies to be included.

## Results

The comprehensive literature search found five HIV prevention programs for young IDUs that met the criteria as outlined above (see Additional file 1). More than fifty studies were considered and five met the criteria to be included in the study. The programs included were from Australia [7,8] and the United States [9-11]. All five studies reported favourable HIV-related outcomes, although inspection of Table 1 reveals the following discrepancies between the studies:

All five studies aimed to reduce risk behaviours or decrease incidence of HIV and other BBVIs. Biological outcomes such as an objective measure of HIV/HCV sero-status were absent in these studies. The outcome measured of risk behaviour varied. One outcome measure was *BBVI knowledge*, which was measured by a questionnaire pre and post-intervention [8] as well as by evaluation feedback questionnaires [7]. A second outcome measure was *injecting risk practices*, which was measure by self-report questionnaires [8-10]. Each study employed differing measures of injecting risk behaviours, such as using a new needle/syringe at last injection, the number of sharing partners and the frequency of needle/syringe sharing and other injecting equipment sharing. A third outcome measure used in two studies was *sexual risk practices*, measured by self-report questions. A fourth outcome used was following through with *HIV-related health referrals*, measured by using self-report questions.

Further outcome measures were used as indicators of a successful program rather than outcomes directly related to changes in risk behaviours. Firstly participants' satisfaction with the program was measured using focus groups [7, 11 evaluation sheets [7], in an open-ended interview [8,11]] and a structured interview [8]. Participants' perception of the program's impact was measured by focus groups and evaluation sheets [7] and structured and open-ended interviews [8]. Lastly, service utilisation was measured. Participants' use of Harm Reduction Central's services such as the needle exchange service was used as an outcome measure by [11]. Service utilisation could also be an indirect measure of reduced syringe sharing; if participants were using the needle exchange services to a greater extent then they were likely to be sharing needles to a lesser extent. Gleghorn et al. [9] included a measure of Outreach Worker contact to determine the effect of different levels of contact on injecting and sexual risk behaviours.

The target population varied in each of the HIV program studies. Firstly the definition of a young person varied, from under 26 years old to 12–23 years. However the most common definition of youth in the literature is between the ages of 15 and 24 years [4] although this definition was used in only one program study [7]. Secondly the definition of an injecting drug user varies, from those at risk of commencing injecting to injecting more than once a week. Thirdly the typical target population varied, with some programs targeting specific cultural and socio-economic subsets of the young IDU population, resulting in difficulties in comparing the effectiveness between the studies, as certain studies may vary in effectiveness depending on the target population.

The sample sizes of the studies varied from 13 to 1,146 young IDUs. Sample size will impact on the statistical power of the study. Small sample sizes are unlikely to allow differences to be detected. Also, the assessment periods of the studies differed. Three studies were cross-sectional [9-11] whereas others were longitudinal [7,8]. One cross-sectional study [9] conducted measures pre-intervention at the intervention site and at a comparison site. Measures were then conducted during the program (which was a continuous outreach program) at the program site and two other comparison sites. One cross-sectional study conducted measures during the program at the intervention site and a comparison site only, and another cross-sectional study conducted measures during the program but without a comparison group. The study conducted by Sheaves et al. [8] conducted measures pre and post-intervention and at one-month follow-up but was without a comparison group. The study conducted by Maher et al. [7] conducted assessment post-intervention and at 2-week follow-up, but was without baseline data and a comparison group.

## Conclusion

The United Nations aims to reduce HIV prevalence among 15–24 year olds by a quarter in the most affected countries by 2005 and globally by 2010 [1]. For this to be possible, youth require easy access to a wide range of effective HIV prevention programs. They require information and skills to help them adapt and maintain behaviours that are protective against HIV infection.

The studies presented in Table 1 employed disparate methodologies, making a comparison of relative effectiveness impossible. According to the authors, all studies had some positive benefit for reducing HIV-related outcomes in the sample, whether it be increasing participant knowledge of HIV or other BBVIs or reducing needle and syringe sharing. However, they all differed in outcomes measured, instruments used, target population and study design. Due to differences in the programs' nature and length and other practical constraints such as budgetary factors, it is unrealistic to expect a good level of consistency. Nonetheless, it is not unrealistic to expect better consistency than that presented in Table 1 (see additional file 1). As outlined in the Introduction, standardisation is now recognized as crucial and is in urgent need of implementation. The need for standardisation still needs to be emphasised, as standardized program findings are critical to informing and guiding appropriate prevention responses [6]. Moreover, the push for standardized indicators which can be compared across countries is necessary in order to determine which programs are effective in particular settings. Furthermore it should be noted that all the studies in Table 1 have come from research based in the US or Australia. It is unclear whether the results from these countries can be applied to other countries.

The studies presented in Table 1 only measured behavioural outcomes and did not contain biological data. Biological data (HIV seroincidence for example, conducted by [12]) are an objective way of determining the effects of a prevention program. However, conducting a large-scale study with HIV serology, large enough to detect a noticeable effect between the intervention and control group would be costly and may not be feasible. Other objective data that could be used as outcome measures are service utilization measures such as those used by Weiker et al. [11]. It could be inferred, for example, that an increase in syringe distribution at a Needle and Syringe Program may be an indicator of a reduction in needle sharing or a reduction in the time syringes remain in circulation. As self-report data have the problems of recall bias and social desirability, combining these data with biological and/or service utilization data would improve the evidence of the effectiveness of a prevention program.

The studies presented in Table 1 additionally lack cost-benefit data, crucial information to aid policy makers and program managers in allocating resources, particularly in resource poor settings. Random sampling was not conducted in any of the studies, thus biases in the results may exist. Although random sampling of individuals in one location for example may not be feasible, random allocation of the intervention to certain clinics may be one solution. Two studies did conduct assessments at an intervention site as well as one or more comparison sites, although these studies did not measure the same participants pre and post intervention, and so were unable to determine the effect of the prevention program on HIV-related outcomes. These short comings resulted in less conclusive data. However the desire to obtain accurate information and the costs and practical issues in collecting such information need to strike a balance and although the ideal should be strived for, it is not always possible. Another reason for the lack of research on youth has been the restrictions imposed by many ethics committees on researchers accessing young people.

UNAIDS proposes that HIV prevention programs for youth should have the following characteristics for effectiveness:

• To succeed they should respect and involve young people, while being sensitive to their cultures.

• Young people need a safe and supportive environment, with sensitive attitudes, policies and legislation at family, community and national levels.

• The stigma and discrimination associated with HIV/AIDS needs to be diffused.

• Strong and effective education systems are important, yet in many countries education systems are clearly in disarray.

• Outreach and peer education programs among young drug users should be expanded, and include steps to improve access to information and prevention equipment such as condoms and needles and syringes and HIV/AIDS care services.

Upon searching the literature for recommendations of standardized instruments and study designs to integrate in a new evaluation study of a HIV prevention program, no clear guidelines were found. If standardised, valid and reliable instruments were in the literature, then program managers would be more likely to adopt them in practice. An important confounding concern is that although standardized indicators are needed that can be compared across countries and regions of the world, failure to adapt these indicators to the local setting can weaken our ability to obtain valid information. Key experts in the field need to make clear recommendations of evaluation study design and instruments to use, preferably at a global level. The following recommendations are a result of this review, with respect to study design, outcomes and instruments to use:

(1) Randomise (at clinic level or individual level)

(2) Collect baseline, post-intervention and follow-up data on the same clients

(3) Use control or comparison group

(4) Collect behavioural and biological data

(5) Use standardized instruments to measure HIV outcomes.

## Competing Interests

The author(s) declare that they have no competing interests.

## Supplementary Material

Additional File 1 Summary of Studies of programs for young and new injecting drug users Click here for file

## References

[B1] United Nations Children's Fund & Joint United Nations Programme on HIV/AIDS and World Health Organisation (2002). Young People and HIV/AIDS Opportunity in Crisis: United Nations Children's Fund (UNICEF).

[B2] Ball A (2000). Adolescents, Substance Use, and HIV/AIDS: Implications for International Research. Paper presented at the Third Global Research Meeting on HIV Prevention in Drug-Using Populations in Durban, South Africa.

[B3] Kleinman PH, Goldsmith DS, Friedman SR, Hopkins W, Des Jarlais DC (1990). Knowledge about and behaviors affecting the spread of AIDS: a street survey of intravenous drug users and their associates in New York City. International Journal of the Addictions.

[B4] World Health Organisation International Collaborative Group (1994). Multi-City Study on Drug Injecting and Risk of HIV Infection.

[B5] Centers for Disease Control and Prevention & AIDS Community Demonstration Projects Research Group (1999). Community-level HIV intervention in 5 cities: final outcome data from the CDC AIDS community demonstration projects. American Journal of Public Health.

[B6] Siushanian JA, Archibald CP, Weiler GA (2002). HIV prevention among injection drug users: the development of indicators to monitor and inform public health action. 11th Annual Canadian Conference on HIV/AIDS research.

[B7] Maher L, Sargent P, Higgs P, Crofts N, Le T, Kelsall J, Kerger M (2000). Sharing Knowledge to Protect Our Community: A Pilot Program for Research, Risk Reduction and Peer Education with Indo-Chinese Drug Users.

[B8] Sheaves F, Preston P, O'Neil E, Klein G, Hort K (2001). That's SIC: mobilizing peer networks for Hepatitis C prevention. Health Promotion Journal of Australia.

[B9] Gleghorn AA, Clements KD, Marx R, Vittinghoff E, Lee-Chu P, Hatz M (1997). The impact of intensive outreach on HIV prevention activities of homeless, runaway, and street youth in San Francisco: the AIDS evaluation of street outreach project (AESOP). AIDS and Behavior.

[B10] Sears C, Guydish JR, Weltzien EK, Lum PJ (2001). Investigation of a secondary syringe exchange program for homeless young adult injection drug users in San Francisco, California, U.S.A. Journal of Acquired Immune Deficiency Syndromes & Human Retrovirology.

[B11] Weiker RL, Edgington R, Kipke MD (1999). A collaborative evaluation of a needle exchange program for youth. Health Education & Behavior.

[B12] Des Jarlais DC, Casriel C, Friedman SR, Rosenblum A (1992). AIDS and the transition to illicit drug injection- results of a randomised trial prevention program. British Journal of Addiction.

