# Supplementary material for: A review of HIV prevention among young injecting drug users: A guide for researchers
Source: Harm Reduct J. 2005 Mar 17;2:5. doi: 10.1186/1477-7517-2-5 (PMC1082909; doi:10.1186/1477-7517-2-5)
Supplement: Additional File 1 — Summary of Studies of programs for young and new injecting drug users [file 1477-7517-2-5-S1.doc]

| **Table 1 Summary of studies of programs for young and new injecting drug users** | | | | | | | | |
| --- | --- | --- | --- | --- | --- | --- | --- | --- |
| Project | Target population | Program | Intervention duration | Sample size | Evaluation measures | Assessment periods | Evaluation findings |  |
| SIC project (Safer Injecting Cwiz)  1999-2000  (Sheaves et al., 2001) | < 26 yrs  Peer networks  IDU or at risk of IDU | Educational session on Hep C risks & transmission | 1 session | 219  baseline  95  follow-up | Injecting risk practices  (self-report)  HCV knowledge (SIC quiz)  Satisfaction and perceived project impact  (Structured and open-ended interview) | Pre-intervention  Post-intervention  1 mo. follow-up  No control | Reduction in:  -needle/syringe sharing  -other injecting equipment sharing  -no. sharing partners  Increase in HCV knowledge  High satisfaction with project |  |
| The Sharing Knowledge to Protect our Community pilot project 1999 (Maher et al., 2000) | 15-24 yrs  Indo-Chinese | Peer-educator sessions on BBVIs, safer drug use & interpreting test results | 12 sessions  over 2 weeks | 13 Heroin  users | BBVI knowledge  Satisfaction and perceived project impact  (Evaluation sheets after training and focus group 2 weeks after training) | Post-intervention  2 week follow-up  No baseline  No control | Increase in self-report of BBVI knowledge  Satisfaction with training  Satisfaction with educating peers and parents  Additional training time required due to low education level |  |
| San Francisco Outreach Program 1993-1995  (Gleghorn at el., 1997) | 12-23 years  Street youth | Free resources (eg.condoms)  HIV prevention activities (discussions, group activities)  Subculture-specific activities and materials  A separate youth NEP set up in area during intervention | From 1993 to 1995 | 1,146  homeless  youth | Using new needle/syringe  Following through with HIV-related health referrals  Condom use, main and casual partner  (Standardized self-report questionnaire) | 6 data sets  Pre-intervention:  -Intervention site  -Comparison site  During intervention  -Intervention site  -3 comparison sites  Cross-sectional | Increase in Outreach Worker contact associated with:  -following through HIV- related  referrals  -new needle/syringe at last injection  (5 times as likely)  Increase of new needle/syringe use at last injection (3 times as likely)  No change in condom use |  |
| Peer Run Secondary SEP, 1997  (Sears et al., 2001) | 15-25  Homeless | Exchange service (syringes, cookers, cotton, etc)  Subculture-specific activities and materials  4 core peer leaders & staff  24 hour service aim | From 1996 to 1997 | 122  homeless  youth | HIV injection and sexual risk behaviors (structured interview) | During intervention  -Intervention site  -Comparison site  Cross-sectional  No baseline | Reduction in  -Syringe sharing  -Syringe reuse  -Sharing cotton to filter drugs  -Inconsistent condom use with  casual partner  No Change in  -Frequency of condom use with  main partner  -Backloading (dividing drugs) |  |
| Needle Exchange Program in LA, 1996 (Weiker et al., 1999) | All youth | Storefront facility called HRC (Harm Reduction Central)  Needle exchange and distribution  Safer shooting kits and information (bleach, cotton, cookers, condoms)  Youth-specific activities  Counseling & case management | From 1993 to 1996 | N Not  present e ed | Use of HRC services and risk behavior (Structured quantitative interviews)  Satisfaction and perceived project impact (focus groups & individual open-ended interviews) | During intervention  Cross-sectional  No baseline  No control | Increase in outreach staff/youth involvement  Needle exchange used three times more frequently than other program components  Youth found HRC a safe non-judgmental place to seek services  Peer staff role was crucial in engaging youth |  |
